# Gram-Negative Bloodstream Infections in Healthcare: The Relationship Between Antibiotic Resistance, Mortality, and Novel Serological Biomarker

**DOI:** 10.7759/cureus.57720

**Published:** 2024-04-06

**Authors:** Nilgün Altın, Can Huseyin Hekimoğlu, Tülay Unver Ulusoy, Semanur Kuzi, Ganime Sevinç, Asiye Tekin, Begum R Aksoy, Irfan Şencan

**Affiliations:** 1 Infectious Diseases and Clinical Microbiology, Ankara Etlik City Hospital, Ankara, TUR; 2 Infection Prevention and Control Unit, Ministry of Health General Directorate of Public Health, Ankara, TUR; 3 Infection Control Committee, Ankara Etlik City Hospital, Ankara, TUR; 4 Infectious Diseases and Clinical Microbiology, Ankara Etlik city Hospital, Ankara, TUR

**Keywords:** novel serological biomarker, mortality, antimicrobial resistance, hospital-acquired, bacteremia, bloodstream infection

## Abstract

Background: Bloodstream infections caused by Gram-negative bacteria are highly mortal. In this study, we aimed to investigate the relationship between antimicrobial resistance profile and novel serological biomarkers and mortality in bloodstream infections (BSIs) caused by Gram-negative bacteria in intensive care units (ICUs).

Methods: 366 Patients diagnosed with healthcare-associated Gram-negative bloodstream infection in the ICUs of our hospital between February 2015 and December 2021 were included in the study. Demographic variables (age, gender, comorbidities), causative microorganisms and antimicrobial susceptibilities, time to first positive blood culture after hospitalization, length of stay in hospital, surgical procedures, laboratory data (hemograms, C-reactive protein (CRP) levels, albumin), and survival data were collected. Novel serological biomarkers were calculated.

Results: Mortality in Gram-negative bloodstream infection was found to be associated with age and novel serological biomarkers, but not with carbapenems and colistin minimum inhibitory concentration (MIC) values. Mortality rates increased with age (p˂0.001). Patients who died had higher C-reactive protein/albumin ratio (CAR) (p<0.001) and neutrophil/lymphocyte ratio (NLR) (p=0.009) and lower prognostic nutritional index (PNI) (p<0.001).

Conclusion: The study emphasizes that resistance to colistin and carbapenems is not associated with mortality in BSIs caused by Gram-negative bacteria. Novel serological biomarkers may be useful in predicting mortality. These results support the need for further studies to elucidate the true impact of infections caused by resistant bacteria.

## Introduction

The treatment opportunities are narrowing and becoming more difficult with the increasing antimicrobial resistance in the world. The increase in resistance rates leads to prolonged hospitalization of patients in multidisciplinary units and increased treatment costs, morbidity and mortality rates [1]. Healthcare-associated infections (HAIs) are a major problem in intensive care units (ICUs). Bloodstream infection is the most common HAI seen in ICUs and is associated with the highest mortality rates (25-66%) [2]. Gram-negative bacteria are often resistant to multiple drugs and are increasingly resistant to most available antibiotics [3]. In this patient group, treatment options have narrowed due to resistance, which is why they are becoming increasingly important worldwide. Measures to prevent the spread of resistance among a wide range of drug-resistant bacterial species (especially carbapenem-resistant Gram-negative bacteria, including carbapenem-resistant Enterobacteriaceae (CRE) and carbapenem-resistant *Acinetobacter baumannii* (CRAb)) are important. Carbapenems are antibiotics of last resort for them; therefore, infectious diseases caused by bacteria resistant to this drug are difficult to treat. In order to reduce the threat of antimicrobial resistance, it is very important to establish an evidence-based information system through surveillance. Surveillance is successfully carried out in Turkey through both national (NIHIESA) and World Health Organization-integrated (CAESAR) surveillance networks [3,4].

In this study, we aimed to investigate the relationship between antimicrobial resistance profile and new biomarkers and mortality in bloodstream infections (BSIs) caused by Gram-negative bacteria in ICUs.

## Materials and methods

Study population

Patients over the age of 18 years who were followed up with a diagnosis of healthcare-associated Gram-negative bloodstream infection (HA-GNBSI) in the nine ICUs (isolation ICU, internal medicine ICU, cardiovascular surgery ICU, surgery ICU, anesthesia and reanimation ICU, neurology ICU, neurosurgery ICU, COVID-19 ICU) of our hospital between February 2015 and December 2021 were included in the study. The total number of beds in these ICUs was 68. Cases were defined as any patient with ≥1 positive blood culture for Gram-negative bacteria 48 hours after ICU admission. Patients were identified according to the Centers for Disease Centers (CDC) criteria [3,5]. Patients under 18 years of age, patients who stayed in ICU for less than 48 hours, patients without adequate medical records, patients with hematologic malignancy and organ transplantation, and immunodeficient and neutropenic patients were excluded.

Data sources and measurement

The infection control committee is actively working in our hospital and active prospective patient-based HAI surveillance is performed by certified infection control nurses. Surveillance is performed according to national diagnostic criteria adapted from the CDC. Bloodstream infections are also monitored by laboratory-based passive surveillance. Records are reported to a national online software network (NIHIESA).

Patient data were obtained from infection control committee records and record electronic systems of the hospital. Demographic variables (age, gender, comorbidities), causative microorganisms and antimicrobial susceptibilities, time to first positive blood culture after hospitalization, length of stay in hospital, surgical procedures, laboratory data (hemogram, C-reactive protein (CRP) levels, albumin), and survival data were collected.

Novel serological biomarkers were calculated. These markers are composed of two or more clinical laboratory indicators. The novel biomarker and their formulas are as follows; CAR = CRP (C-reactive protein) (mg/L) /albumin(g/L), NLR = neutrophil counts (10^9^/L) /lymphocyte counts(10^9^/L), PNI (Prognostic nutritional index) = 10 × albumin(g/L) + 5 × lymphocyte count (10^9^/L), PLR (platelet-to-lymphocyte ratio) = platelet counts (10^9^/L) /lymphocyte counts (10^9^/L), SII (systemic immune inflammation index) = platelet count (10^9^/L) × neutrophil count (10^9^/L)/lymphocyte count (10^9^/L) [6].

Microbiologic studies

Blood culture isolates that were eligible according to CDC criteria were included in the study [5]. The four most common microbiologic agents in the ICUs in Turkey were considered [4]. Identification and minimum inhibitory concentration (MIC) determination of the microorganisms were conducted using the automated system (VITEK 2, Biomerieux, Marcy l’Etoile, France, or Phoenix, BD, Franklin Lakes, NJ, USA). Antibiotic susceptibilities were determined according to the European Committee on Antimicrobial Susceptibility Testing (EUCAST) criteria [7]. All microbiology laboratories have been following EUCAST standards since 2014. Colistin susceptibility was analyzed by the microtube dilution method. According to EUCAST, meropenem, imipenem, and colistin susceptibilities were analyzed. Sensitive (S), Resistant (R), or Intermediate (I) susceptibility of antimicrobials was based on MIC levels. [7]. The relationship between carbapenems and colistin resistance status of the four most common microorganisms with mortality after ICU admission was evaluated.

Ethical approval

This study has received ethical approval from the Ankara Etlik City Hospital Ethics Committee (approval no: AESH-EKI-2023-326, approval date: 12/07/2023).

Statistical analysis

Descriptive statistics are summarized with means and standard deviations, numbers, and percentages. The 95% confidence interval for percentages was calculated with the “Score (Wilson)” method. The chi-square test was used for the comparison of categorical variables. Continuous data were compared by independent samples t-test when appropriate. The significance threshold (p-value) was set at 0.05 for all tests. Graphs were generated using the program Excel. The OpenEpionline program was used for the statistical analysis of the data. (https://www.openepi.com/Proportion/Proportion.htm).

## Results

HA-GNBSI was detected in a total of 366 patients and a total of 391 BSIs. Of these, 278 (76.0%) were related to central catheterization and 152 (41.5%) to surgical intervention. One hundred ninety-four (53.0%) of the total patients were male. Mean age was 65.1 years (SD 17.7), mean length of hospitalization was 39.4 days (SD 43.2), mean duration of infection was 27.7 days (SD 50.0). While the length of hospitalization and development of infection was not different between men and women (p=0.109, p=0.132), female age (67.1 ± 17.7) was statistically significantly higher than male age (63.39 ± 17.58) (p=0.043). The mean age was 57.3± 18.4/year in discharged patients and 67.3± 16.8/year in exitus patients. The mean length of hospitalization was 46.8± 38.6/day for those discharged and 37.4 ±44.2/day for those who died. The mean duration of infection development was 20.5 ±18.1/day in those discharged, and 22.2± 34.4/day in those who died.

The fatality rate was high and 285 (78.0%) of the patients died. We investigated the association of that with HA-GNBSI. Of the blood cultures, 380 (97.2%) were mono-microbial and 11 (2.8%) contained two different microorganisms. The four most common Gram-negative bacteria included in the study were *A. baumannii* 194 (48.3%), *Klebsiella pneumoniae* 117 (29.1%), *Escherichia coli* 55 (13.7%), and *Pseudomonas aeruginosa* 36 (9.0%).

**Figure 1:**
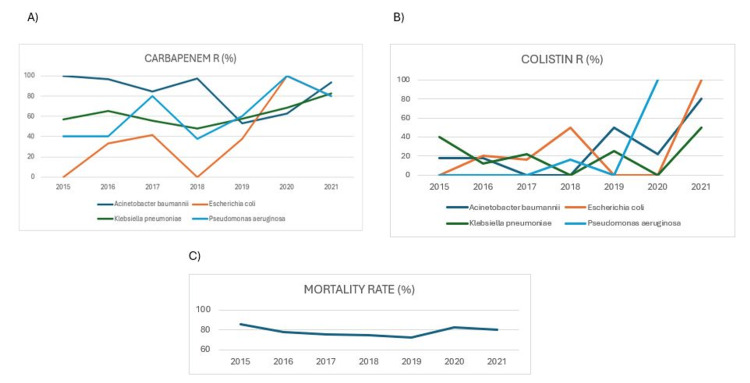
Changes in carbapenem and colistin resistance (A, B), and total mortality rates over the years (C).

According to laboratory records, when classified according to antimicrobial susceptibility without considering MIC values, the overall resistance rates to colistin and carbapenems (meropenem, imipenem) were as follows: For *A. baumannii*, 14 of 80 strains were colistin-resistant (17.5%), 158 of 193 strains were carbapenem-resistant (81.9%); for *K. pneumoniae*, nine of 54 strains were colistin-resistant (16.7%), 71 of 115 strains were carbapenem-resistant (61. 7%); for *E. coli*, six of 26 strains were colistin-resistant (23.1%), 21 of 54 strains were carbapenem-resistant (38.9%); for *P. aeruginosa*, two of 22 strains were colistin-resistant (10.0%), 21 of 36 strains were carbapenem-resistant (58.3%). Although resistance rates increased over the years, there was no significant change in the average mortality rate (Figure 1).

**Figure 2:**
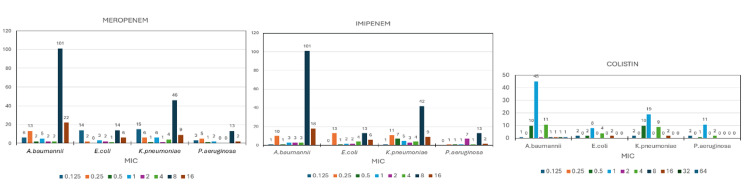
Distribution of MIC values of microorganisms according to antimicrobial. MIC: minimum inhibitory concentration

The distribution of MIC values of microorganisms according to antimicrobial are presented in Figure 2. The resistance rates of Gram-negative microorganisms when carbapenem and colistin susceptibilities were classified according to MIC values are given in Table 1. There was no significant correlation between MIC values and mortality (Table 1). Although it was not statistically significant, the mortality rate of meropenem intermediate susceptible *E. coli* was high at 93.3% (95% CI: 70.2-98.8). And meropenem-resistant *A. baumannii*, *P. aeruginosa*, and *K. pneumoniae* also had high mortality rates of 81.8% (95% CI: 61.5-92.7), 100% (95% CI: 34.2-100.0), 88.9% (95% CI: 56.5-98.0), respectively (Table 1).

**Table 1:** Antimicrobial susceptibilities of Acinetobacter baumannii, Klebsiella pneumoniae, Escherichia coli, and Pseudomonas aeruginosa strains according to MIC-based classification and their relationship with mortality were evaluated. CI and p values are given. MEM: meropenem, IMP: imipenem, COL: colistin, EX: exitus, CI: confidence interval

Causative microorganism	Antibacterial sensitivity	Discharge	Ex (n,%)	95% CI	p-value	Total
*Acinetobacter baumannii*	MEM S	10	18 (64.3)	45.8-79.3	0.1129	28
	MEM I	26	77 (74.8)	65.6-82.1		103
	MEM R	4	18 (81.8)	61.5-92.7		22
	IMP S	6	12 (66.7)	43.7-83.7	0.3230	18
	IMP I	1	2 (66.7)	20.8-93.8		3
	IMP R	29	90 (75.6)	67.2-82.5		119
	COL R	2	8 (80.0)	49.0-94.3	0.8913	10
	COL S	13	44 (77.2)	64.8-86.2		57
*Escherichia coli*	MEM S	3	18 (85.7)	65.4-95.0	0.2481	21
	MEM I	1	14 (93.3)	70.2-98.8		15
	MEM R	1	5 (83.3)	43.5-97.0		6
	IMP S	2	16 (88.9)	67.2-96.9	0.7575	18
	IMP I	1	3 (75.0)	30.1-94.4		4
	IMP R	2	17 (89.5)	68.6-97.1		19
	COL R	1	3 (75.0)	30.1-95.4	0.7912	4
	COL S	2	9 (81.8)	52.3-94.8		11
*Klebsiella pneumoniae*	MEM S	6	23 (79.3)	65.6-90.1	0.6954	29
	MEM I	13	37 (74.0)	60.4-81.1		50
	MEM R	1	8 (88.9)	56.5-98.0		9
	IMP S	6	21 (77.8)	59.2-89.4	0.7100	27
	IMP I	3	1 (25.0)	4.6-96.9		4
	IMP R	11	40 (78.4)	65.4-87.5		51
	COL R	2	5 (71.4)	35.9-91.8	0.8261	7
	COL S	10	30 (75.0)	59.8-85.8		40
*Pseudomonas aeruginosa*	MEM S	3	8 (72.7)	43.4-90.3	0.4488	11
	MEM I	4	9 (69.2)	42.4-87.3		13
	MEM R	0	2 (100.0)	34.2-100.0		2
	IMP S	3	8 (72.7)	43.4-90.2	0.9149	11
	IMP I	4	11 (73.3)	48.1-89.1		15
	IMP R	3	7 (70.0)	39.7-89.2		10
	COL R	0	2 (100.0)	34.2-100.0	0.8674	2
	COL S	4	9 (69.2)	42.4-87.3		13

The study analyzed mortality by univariate factors and included 337 patients with a single infection with a single agent. Patients with multiple agents and infections were not included in the analysis. There was no difference in mortality according to gender (p=0.134), type of ICU (p=0.312), Central line-associated BSI (CLA-BSI) (p= 0.653), surgery procedure (p=0.500) type of causative microorganism (p=0.626), infection development time (p=0.566), and length of hospitalization (p=0.087). While mortality rates increased with age (p˂0.001), no difference was found between male-female gender (p=0.134), and exitus (Table 2). Among the causative microorganisms, *P. aeruginosa* (28.6% [95% CI: 52.9-74.6]) was the most common cause of death (p=0.626) (Table 2).

**Table 2:** Mortality was evaluated by univariate analysis. ICU: intensive care unit; CLA-BSI: central line-associated bloodstream infection; LC-BSI: lab-confirmed bloodstream infection; AR-ICU: anesthesia and reanimation intensive care unit; IM-ICU: internal medicine ICU; CS-ICU: cardiovascular surgery ICU; BS-ICU: brain surgery ICU; Ex: exitus; CI: confidence interval

Patient characteristics	Variable	Discharged	Ex (n, %)	95% CI	p-value	Total
Gender	Male	37	157(80.9)	74.8-85.8	0.1344	194
	Female	44	128(74.4)	67.4-80.4		172
Type of ICUs	AR-ICU	36	121(77.1)	69.9-82.9	0.3120	157
	Surgery ICU	7	32(82.1)	67.3-91.0		39
	COVID-19 ICU	6	27(81.8)	65.6-91.4		33
	IM-ICU	3	11(78.6)	52.4-92.4		14
	Isolation ICU	3	18(85.7)	65.4-95.0		21
	CS-ICU	9	41(82.0)	69.2-90.2		50
	Neurology ICU	5	18(78.3)	58.1-90.3		23
	BS-ICU	12	17(58.6)	40.7-74.5		29
Source of HA-BSI	CLA-BSI	21	67(76.1)	66.3-83.8	0.6533	88
	LCA-BSI	60	218(78.4)	73.2-82.8		278
Surgery procedure	Performed	31	121(79.6)	72.5-82.2	0.5001	152
	None	50	164(76.6)	70.5-81.8		214
Causative microorganism (Gram-negative bacteria)	*A. baumannii*	38	130(22.6)	70.5-83.1	0.6262	168
	*E. coli*	7	32(17.9)	67.3-91.0		39
	*K. pneumoniae*	19	83(18.6)	72.7-87.7		102
	*P. aeruginosa*	8	20(28.6)	52.9-74.6		28

Patients who died in the hospital and who survived were also compared with respect to the novel serological biomarker levels. Patients who died had a higher C-reactive protein/albumin ratio (CAR) (p<0.001) and neutrophil/lymphocyte ratio (NLR) (p=0.009) and a lower prognostic nutritional index (PNI) (p<0.001) than survivors (Table 3). 

**Table 3:** Mortality evaluated with novel serological biomarkers. CAR: C-reactive protein/albumin ratio; CRP: C-reactive protein; ALB: albumin; PNL: neutrophil; LYM: lymphocyte; PLT: platelet; SII: systemic immune inflammation index; PNI: prognostic nutritional index; NLR: neutrophil/lymphocyte ratio; PLR: platelet-to-lymphocyte ratio

	Exitus			Discharge			p-value
	n	average	Sd	n	average	Sd	
CAR (CRP/ALB)	278	86.5	64.2	88	55.7	43.2	0.000000637
NLR (PNL/LYM)	292	21.4	26.3	91	13.2	26	0.009575
PNI (10xALB)+(0,005xLYM)	286	30	7	89	33.9	8.9	0.0002376
PLR (PLT/LYM)	292	0.285	0.325	91	0.323	0.638	0.5859
SII (PLTxPNL/LYM)	292	3795.9	5768.8	91	3715	9933.6	0.9412

## Discussion

In this study, we found that mortality in GNBSI was not associated with carbapenems and colistin resistance, but was significantly associated with age and novel serological biomarkers CAR, NLR, and PNI. BSIs caused by resistant bacteria were compared with infections caused by susceptible bacteria and mortality rates were similar. There was no correlation found between the increase in resistance rates and mortality rates according to 6-year data.

Infection is an independent risk factor for mortality in ICU patients. In addition, HAIs have significant effects on morbidity and length of hospitalization [8]. Although there are studies claiming that mortality is affected by increasing resistance rates of bacteria, this issue is yet unclear. In a review on this issue, 24 articles were examined, and no relationship was found between resistance and mortality in 10 articles, while inappropriate antibiotic treatments and methodological reasons were emphasized in the other articles [9]. Bonnet et al. had compared mortality rates in Gram-positive and Gram-negative bacteria and in contrast to this study, the mortality rate was significantly higher in Gram-negative bacteria [8]. In a study of 1556 cases evaluating data from different centers over a period of approximately two years, increasing MIC values of both carbapenems and colistin were independently associated with higher case fatality race [10]. This result may have been affected by the different treatment approaches of different centers and antimicrobial resistance changes were not adequately observed over the short period of two years.

Increasing rates of resistance limit treatment options. In a previous study, it was reported that ICU-BSI was associated with a 40% increase in the risk of 30-day mortality, especially if early antimicrobial treatment was not adequate [11]. This demonstrates that with the accelerating rates of resistance, the mortality rate may increase if appropriate antibiotics are not available [11]. The literature suggests that the risk of death for ICU-BSI due to multidrug-resistant pathogens or susceptible pathogens is similar when treated promptly and with appropriate antimicrobial agents [8]. Resistance rates are gradually increasing and multidisciplinary intervention is necessary to prevent this. In this issue, it is very important to establish common approach strategies and training, rapid implementation of diagnosis and treatment by considering resistance possibilities, correct and sufficient duration of antibiotic use, and resource control [8,12-16].

The advantage of our hospital is that patient treatments are organized by a single clinic and therefore the treatments are similar. New-generation antibiotics other than meropenem, imipenem, and colistin were not used in our hospital until 2021. With surveillance data, the right choices are made in empirical treatment and when a blood culture signal is received by microbiology, antibiotic treatments are initiated without delay.

Increasing age is an important risk for HAIs; in the elderly population, the immune system works less efficiently, so the risk of contracting HAIs increases. In addition, the elderly population often has comorbidities that affect immunity [8]. For these reasons, age is an important factor associated with mortality. The mean age of the patients followed up in the ICU was 65.1 years, and consisted of an elderly population. The statistically significant difference of elder age in mortality between patients who died and those who survived was an expected result.

Novel serological biomarkers can be easily calculated by routine blood tests and can be used to identify patients at high risk of mortality. In this study, CAR and NLR were significantly higher in patients who died. There are not enough studies on this subject. Li et al. found that the novel biomarkers CAR, NLR, and PNI were significantly altered in patients with invasive candidiasis. These biomarkers were significantly associated with disease severity and prognosis of ICU patients [6]. PNI is a nutritional index. In a previous study, PNI was associated with mortality in COVID-19 infection [17]. In another study, a decrease in PNI was found to be associated with the exitus rate in Crimean-Congo hemorrhagic fever [18]. This study investigated the relationship between PNI and mortality in Gram-negative bacterial infections, showing a negative correlation similar to viral infections.

Limitation

The retrospective nature of our study reduces its design power. Molecular or additional biochemical studies could not be performed. We learned the antibiotics the patients received in the ICU and when they were started and discontinued, but not the time of initiation. The history of antibiotic use and hospitalization in the last 3 months was not reported. MIC values for each strain were not available in our data. The number of patients was too small to perform an advanced analysis. However, it also has strengths such as trained and experienced EC nurses, active surveillance system, microbiology laboratory that meets external quality standards.

## Conclusions

Mortality in BSIs caused by Gram-negative bacteria was evaluated. The relationship between colistin and carbapenem resistance and mortality was not significant. It is important to know the factors that increase the mortality of patients for treatment and follow-up strategies. Especially in elderly Gram-negative BSI patients, close follow-up was necessary in terms of mortality. Novel serological markers may be useful in predicting mortality. These results support the need for further studies to elucidate the true impact of infections caused by resistant bacteria.
